# Supplementary material for: Characterization and remediation of sample index swaps by non-redundant dual indexing on massively parallel sequencing platforms
Source: BMC Genomics. 2018 May 8;19:332. doi: 10.1186/s12864-018-4703-0 (PMC5941783; doi:10.1186/s12864-018-4703-0)
Supplement: Supplementary file 1 — Figure S1. Initial testing of single vs dual indexed sequencing on HiSeq. Figure S2. Previous experience with index swapping during exome capture library preparation (previously unpublished data). (DOCX 1113 kb) [file 12864_2018_4703_MOESM1_ESM.docx]

**SUPPLEMENTAL FIGURES**

**Costello M. et. al - Characterization and remediation of sample index swaps by non-redundant dual indexing on massively parallel sequencing platforms**

**Figure S1: Initial testing of single vs dual indexed sequencing on HiSeq.** For four pools of 2 libraries, both the i7 and i5 indexes were read. Demultplexing was then performed two ways, once using just the i7 single index and again using both the i7 and i5 dual indexes. The % contamination rate decreased dramatically when dual indexing demultiplexing was performed.

**
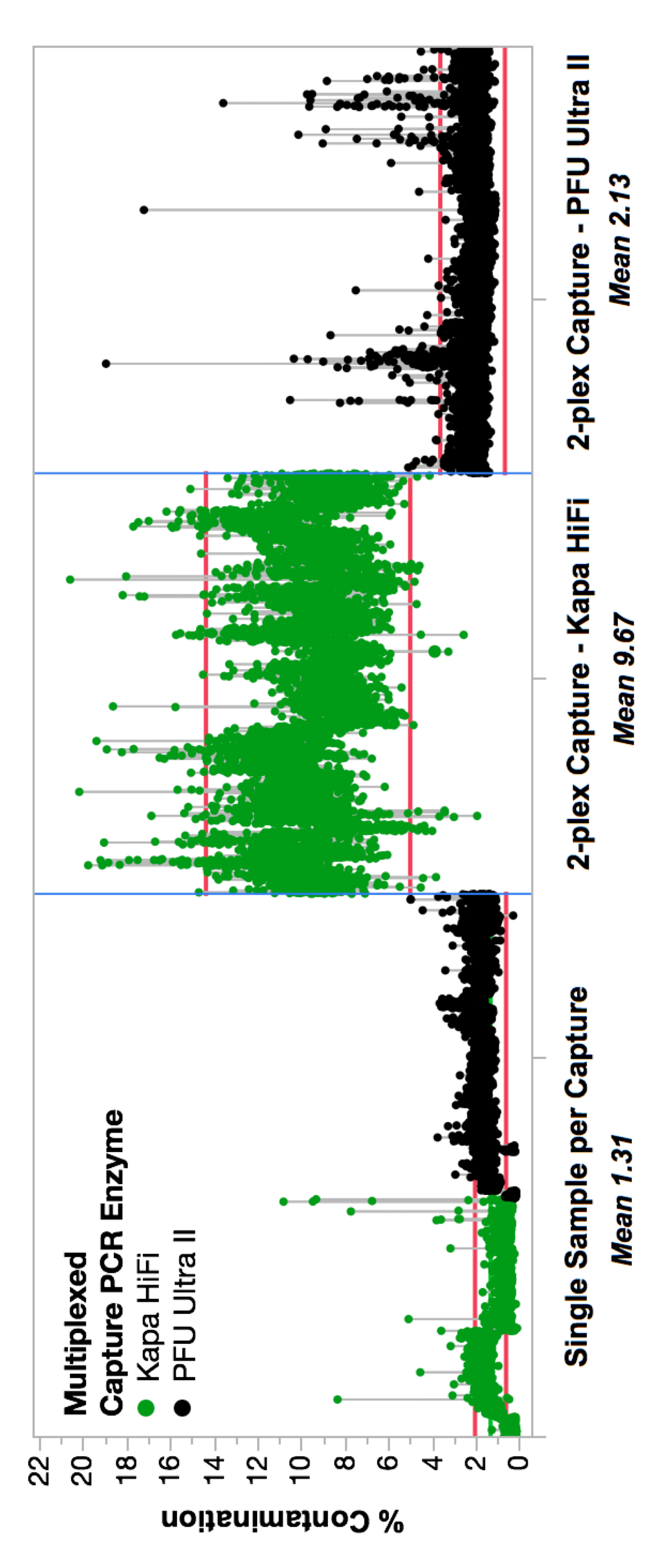
**

**Figure S2: Previous experience with index swapping during exome capture library preparation (previously unpublished data).** Contamination rates for Agilent Sure Select exome capture libraries from 2013 sequenced on HiSeq 2500 (non-ExAmp reagents). We observed a spike in sample contamination when we began to 2-plex our libraries prior to exome hybridization and capture, and saw difference in the severity of the contamination when using different PCR enzymes (Kapa HiFi or PFU Ultra II) during the post-capture multiplex PCR amplification This demonstrates that index swapping can occur anytime samples are amplified together, not just during ExAmp sequencing chemistry.
