# Supplementary material for: Characterization and remediation of sample index swaps by non-redundant dual indexing on massively parallel sequencing platforms
Source: BMC Genomics. 2018 May 8;19:332. doi: 10.1186/s12864-018-4703-0 (PMC5941783; doi:10.1186/s12864-018-4703-0)
Supplement: Supplementary file 3 — Suggested guidelines for designing robust indexes for Illumina sequencing. (DOCX 129 kb) [file 12864_2018_4703_MOESM3_ESM.docx]

**Basic Guidelines for Index Designs for Illumina Sequencing**

Costello M. et al., BMC Genomics

**Background**

While a variety of vendors provide indexing solutions for sample multiplexing prior to sequencing, it may be necessary for individual researchers to design their own set of indexes that meet the needs of their unique projects. While selecting from a list of all possible 8-bp library indexes may seem daunting, we provide here in this document some basic guidelines that can help screen indexes *in silico*.

**Best Practice Guidelines for Index Design**

1. **Ensure 8-bp indexes are more than two errors away from each other within a set (Hamming Distance 3 or greater).** Errors regularly occur during both the i7 and i5 index sequencing reads, and we observe that ~10% of high quality index reads contain 1 or 2 substitution errors. It is therefore extremely important that all indexes within a set are greater than 2 errors away from each other to avoid any index turning into another index in the set due to sequencing errors. This also provides an opportunity to computationally correct indexes with 1 or 2 errors to rescue reads. Alternatively, others have proposed using a Levenshtein Distance screen that not only takes base errors into account, but also frameshifts [1].
2. **i5 indexes should never begin with “AC” as the first 2 bases (ACXXXXXX).** Any index starting with “AC” should not be used in the i5 second index read position due to a known mis-priming issue effecting MiSeq, HiSeq 2500, and NovaSeq. This error mode leads to the first two “AC” bases being skipped, resulting in a frameshifted index read starting at base 3. While HiSeq X, HiSeq 4000, and NextSeq do not have this mis-priming issue, we recommend avoiding “ACXXXXXX” i5 reads altogether to eliminate the need to re-validate new index sets as technologies change.
3. **Avoid repeats of 3 or more of the same base within an index.** Mononucleotide repeats are difficult to sequence on Illumina sequencers. We therefore recommend avoiding indexes that contain repeats of 3 or more of the same base within the 8 bp index (e.g. CTAAAAGT). For NovaSeq and NextSeq, which use 2-color chemistry, it is especially important to avoid runs of G bases.

1. **Perform delta-G self-complementarity analysis on indexes within the adapter or primer sequence to avoid hair pins or secondary structures.** To ensure that indexes do not form hairpins or other secondary structures within the adapter or primer sequences surrounding them, we recommend performing DNA folding analysis using mFold or another program [2]. The suggested delta-G value for any self-complementarity analysis for DNA oligos should be less than (more positive than) -9 kcal/mol [3].
2. **If possible, avoid indexes that are >65% GC or AT.** Higher GC sequences are both harder to amplify by PCR and more difficult to cluster amplify on the flow cell. To create a set of indexes that perform with equal efficiency, we suggest avoiding indexes that are either very high AT or GC (>65%).
3. **Do not use combinatorial or redundant indexes methods.** Because of index swapping in sequencing utilizing ExAmp patterned flow cells, using combinatorial indexing schemes is highly not recommended. This therefore requires validating many more indexes in order to have completely unique i7 and i5 index sets. For 96, this requires a full set of 96 unique i7s and a full set of unique i5s.
4. **Be aware that the oligonucleotide synthesis process may introduce index adapter or primer cross contamination.** When ordering a new set of indexed adapters or primers, we recommend discussing options for ordering with your preferred oligo vendor. They will likely have suggestions for ordering options that could reduce the chance of cross contamination during manufacture.

**References:**

1. Buschmann T, Bystrykh LV. Levenshtein error-correcting barcodes for multiplexed DNA sequencing. BMC Bioinformatics. 2013;14:272. doi:10.1186/1471-2105-14-272.
2. The mFold Web Server. <http://unafold.rna.albany.edu/?q=mfold>. Accessed January 31, 2018.
3. Frequently Asked Questions, Integrated DNA Technologies website. <http://www.idtdna.com/pages/support/how-can-i-check-my-pcr-primers-using-the-oligoanalyzer-program-to-ensure-there-are-no-significant-primer-design-issues->. Accessed January 31, 2018.
